# Supplementary material for: Sequential viral introductions and spread of BA.1 across Pakistan provinces during the Omicron wave
Source: BMC Genomics. 2023 Aug 3;24:432. doi: 10.1186/s12864-023-09539-3 (PMC10399012; doi:10.1186/s12864-023-09539-3)

Supplementary Figure 1. SARS-CoV-2 genome submissions in GISAID from Pakistan. Distribution of 1031 sequences submitted by Pakistan from December 1, 2021 till August 14, 2022. On x-axis are the date in month-year format and y-axis represents the number of SARS-CoV-2 genome submissions in GISAID. Orange circles show the exact number of sequences. *Partial data for August (till August 14, 2022).

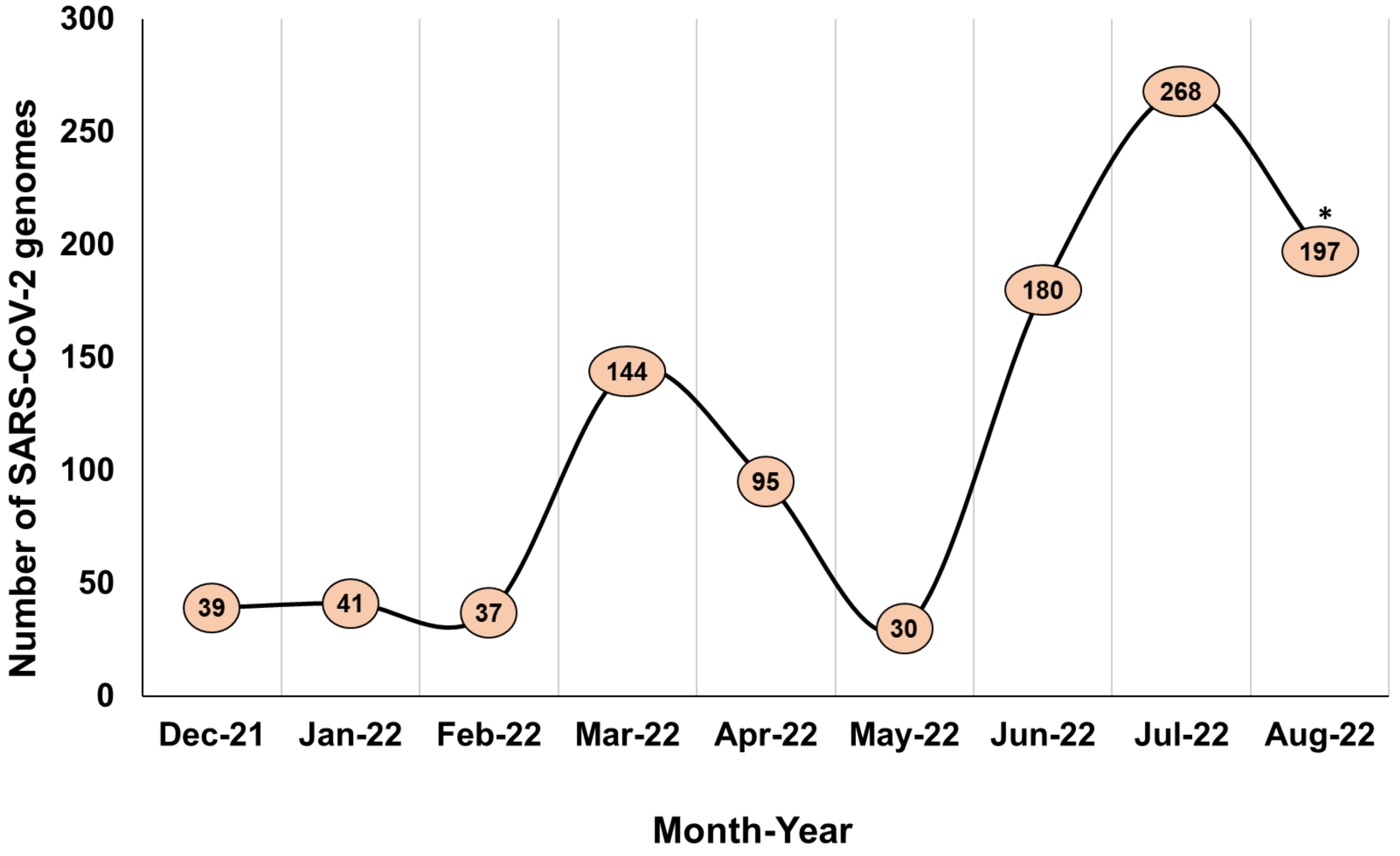

Supplement: Supplementary file 2 — Additional file 2: Supplementary Figure 1. SARS-CoV-2 genome submissions in GISAID from Pakistan. [file 12864_2023_9539_MOESM2_ESM.pdf]
