# Supplementary material for: Sequential viral introductions and spread of BA.1 across Pakistan provinces during the Omicron wave
Source: BMC Genomics. 2023 Aug 3;24:432. doi: 10.1186/s12864-023-09539-3 (PMC10399012; doi:10.1186/s12864-023-09539-3)

Supplementary Figure 2. SARS-CoV-2 genome submissions in GISAID from each region of Pakistan across time. The graph shows all SARS-CoV2 sequences (n=1031) genomes submitted from each region of Pakistan between December 1, 2021 and August 14, 2022. Sindh (light blue), ICT (orange), Punjab (grey), KPK (yellow), Balochistan (blue), GB (green), AJK (dark blue). On x-axis date in month-year format is mentioned and y-axis shows number of SARS-CoV2 genomes submitted in GISAID.

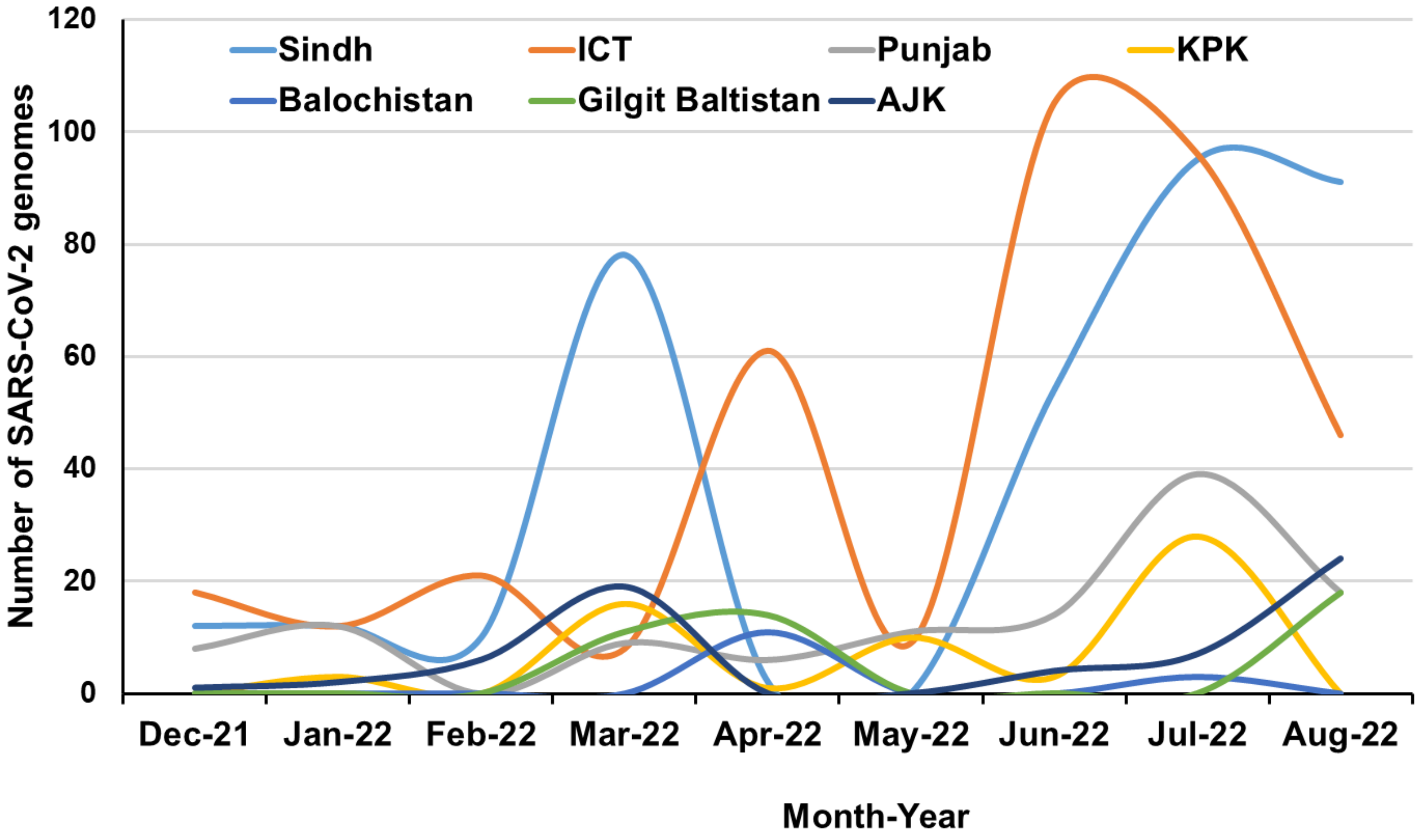

Supplement: Supplementary file 3 — Additional file 3: Supplementary Figure 2. SARS-CoV-2 genome submissions in GISAID from each region of Pakistan across time. [file 12864_2023_9539_MOESM3_ESM.pdf]
